# Evaluation of Maize Genotypes for Northern Leaf Blight Resistance and Yield in Ethiopian Hotspots

**DOI:** 10.1002/pei3.70219

**Published:** 2026-10-15

**Authors:** Mesele Haile, Zelalem Bekeko, Temesgen Deressa, Gemechisa Getachew, Gemechu Alemu

**Affiliations:** ^1^ Ethiopian Institute of Agricultural Research Addis Ababa Ethiopia; ^2^ College of Agriculture and Environmental Sciences Haramaya University Dire Dawa Ethiopia

**Keywords:** AUDPC, disease resistance, Ethiopia, *Exserohilum turcicum*, genotypes, grain yield, heterosis, maize, northern leaf blight, resistance durability

## Abstract

Northern leaf blight (NLB), caused by *Exserohilum turcicum*, is a major constraint to maize production in Ethiopia's mid‐altitude and transitional‐highland agro‐ecologies. This study evaluated 30 maize genotypes (24 commercial hybrids released 1988–2020 and six female parent lines) at two NLB hotspot locations (Arsi Negele, 1960 m a.s.l.; Wondo Genet, 1720 m a.s.l.) during the 2022 and 2023 main cropping seasons. Artificial inoculation using ground‐infected leaf tissue combined with BH543 spreader rows ensured uniform disease pressure. A randomized complete block design with three replications was used at each location. Disease parameters (NLB severity, AUDPC, apparent infection rate, lesion size) and grain yield were assessed. Combined analysis of variance revealed significant genotype × year × location interactions (*p* < 0.001) for all traits, justifying location‐specific analyses. Final NLB severity (NLB6) ranged from 25.1% (F‐BH661) to 53.5% (F‐BH543). Obsolete varieties BH140 (released 1988) and BH540 (released 1995) showed high susceptibility (48.7%–48.8% NLB6), statistically similar to the susceptible check BH543 (51.3%), suggesting possible erosion of resistance effectiveness over 17–27 years, though pathogen race characterization is required for confirmation. Grain yield ranged from 3.2 to 7.9 t ha^−1^, with BH549 achieving the highest yield (6.80 t ha^−1^). Female‐parent relative heterosis analysis revealed contrasting male parent effects: BH661 maintained resistance near the female‐parent level (+6.7% heterosis for NLB6, indicating a marginal increase in susceptibility), while BH546 exhibited substantially increased susceptibility (+53.4%). NLB6 correlated negatively with yield (*r* = −0.40, *p* < 0.01) and positively with AUDPC (*r* = 0.78) and infection rate (*r* = 0.90). F‐BH661 was identified as the most resistant genotype, contributing differentially to BH661 and BH546 resistance. These findings provide critical insights for breeding durable NLB resistance and developing location‐specific variety recommendations for Ethiopian maize production systems.

## 1 Introduction

Maize ( *Zea mays* L.) is the cornerstone of Ethiopian agriculture, serving as the primary staple food crop for over 80 million people and contributing more than 75% of the nation's cereal production (Abate et al. 2015; CSA 2021). The crop's significance extends far beyond subsistence, encompassing livelihoods, income generation, and food security for millions of smallholder farmers across the country's diverse agro‐ecologies. Over the past three decades, Ethiopia has achieved remarkable maize productivity gains, with cultivated area expanding to approximately 2.3 million ha and average yields rising from around 1.6 t/ha in the 1990s to more than 3.7 t/ha recently—driven largely by improved hybrids released by the Ethiopian Institute of Agricultural Research (EIAR) through its network of germplasm testing locations strategically located in major maize‐growing environments (Erenstein et al. 2022; Goshime et al. 2021).

However, this progress faces a persistent and evolving threat from Northern Leaf Blight (NLB), caused by the fungal pathogen *Exserohilum turcicum* (Pass.) Leonard and Suggs, which ranks among the most devastating foliar diseases of maize worldwide, especially in humid, temperate, and tropical highland environments where moderate temperatures (20°C–27°C) and prolonged leaf wetness foster epidemic development (Ohunakin et al. 2023; Onwunali and Mabagala 2020; Haile et al. 2026a) By progressively destroying photosynthetic leaf tissue, the disease compromises grain filling and reduces yields by 15%–50%, with the most severe losses occurring when infection develops before silking (Onwunali and Mabagala 2020; Wende et al. 2013; Haile Onu et al. 2026).

While qualitative resistance governed by major genes often provides high initial protection, its effectiveness can be short‐lived as *E. turcicum* populations evolve through genetic mutation and recombination to circumvent these defenses (Wani et al. 2022). This vulnerability is exacerbated by the loss of effectiveness in formerly resistant cultivars, as evidenced by grain yield reductions between 40.7% and 49.1% reported in susceptible Ethiopian cultivars (Terefe et al. 2023; Haile et al. 2026a). Beyond immediate yield deficits, the disease also compromises seed quality by reducing germination capacity and lowering sugar content, which further predisposes the crop to secondary complications such as stalk rot (Wani et al. 2022).

In Ethiopia, this threat is compounded by the arrival of Maize Lethal Necrosis, which has spread rapidly across East Africa since 2011, often resulting in near‐total yield loss in fields where farmers utilize susceptible commercial hybrids and elite inbred lines (Yadesa and Diro 2023). The presence of these diverse biotic pressures, alongside the persistent risk of Turcicum leaf blight, has necessitated the continuous development of germplasm resistant to multiple fungal diseases within Ethiopia's mid‐altitude sub‐humid agro‐ecologies, where the disease remains more prevalent due to the frequent occurrence of humid weather conditions and moderate temperatures (Gemechu et al. 2018; Keno et al. 2018; Haile et al. 2026a).

In addition to these evolutionary shifts, environmental factors such as agro‐ecological variation and maize farming practices significantly influence the spatio‐temporal distribution and intensity of these epidemics within Ethiopian maize‐producing districts (Haile et al. 2026a). In Ethiopia, NLB has emerged as a particularly critical constraint in designated hotspot areas—regions where environmental conditions consistently favor disease development and where the pathogen has become endemic (Terefe et al. 2023; Tilahun et al. 2012). These hotspots include the mid‐altitude zones around Wondo Genet (1720 m asl) and the transitional highlands around Arsi Negele (1960 m asl), both characterized by high rainfall, moderate temperatures, and intensive, continuous maize cultivation that provides abundant host tissue for pathogen multiplication and survival (Terefe et al. 2023; Tilahun et al. 2012; Haile et al. 2026a). These areas serve not only as major maize production zones but also as natural laboratories where the dynamic interaction between maize genotypes and *E. turcicum* populations can be studied under conditions of high disease pressure (Yu et al. 2008).

The pathogen displays significant genetic diversity, including multiple physiological races that can overcome specific host resistance genes, with studies documenting new races emerging within 5–15 years of resistance gene deployment (Ramathani et al. 2011; Welz and Geiger 2000). This pathogenic variability poses a continuous threat to maize production, as resistance deployed in commercial varieties may be rendered ineffective over time through the emergence of new virulent races—a phenomenon well‐documented in many plant‐pathogen systems (McDonald and Linde 2002a, 2002b). The question of resistance durability—its ability to remain effective over prolonged periods of widespread cultivation—is therefore of paramount importance for sustainable maize production, particularly in hotspot areas where selection pressure on the pathogen population is most intense (Terefe et al. 2023).

These challenges are especially pertinent in Ethiopia, where the national maize breeding program has released numerous hybrids over three decades (1988–2020), many of which have become dominant in farmers' fields (Abate et al. 2015; Marenya et al. 2022; Tarekegne et al. 2024; Daemo et al. 2024). Among these, BH140 (released 1988) and BH540 (released 1995) represent the foundation of the national program's early efforts and were once widely cultivated and considered acceptably resistant. BH543 (released 2005) achieved widespread adoption due to its high yield potential but was subsequently withdrawn from most maize‐producing areas owing to its susceptibility to TLB, leading to catastrophic yield losses in disease‐conducive years (Chivasa et al. 2022; Megersa et al. 2017). The inclusion of these obsolete varieties in the present study provides a unique opportunity to assess whether changes in NLB resistance have occurred over time and to evaluate the evidence for potential resistance breakdown or pathogen evolution.

The critical role of parental lines in determining hybrid resistance adds another layer of complexity to understanding and managing NLB (Hallauer et al. 2010; McDonald and Linde 2002b; Haile et al. 2026b). In hybrid maize breeding, the female parent (seed parent) provides the cytoplasmic genome and half the nuclear genome, while the male parent (pollinator) contributes the other half, and the overall hybrid performance hinges on combining ability and specific allelic interactions that occur in the heterozygous state (Hallauer et al. 2010; McDonald and Linde 2002b). This study evaluates several female parents alongside their commercial hybrids, enabling heterosis assessment (Haile et al. 2026b). Notably, F‐BH661—the female parent of both BH661 and BH546—serves as a key genetic resource, and the contrasting performance of these half‐sib hybrids offers a natural experiment to dissect parental contributions to disease resistance.

Given the dynamic host‐pathogen interplay, Ethiopia's extensive hybrid history spanning three decades, and NLB's critical importance as a production constraint, systematic germplasm evaluation in hotspots is essential. Thus, this study was conducted with the overarching goal of comprehensively evaluating NLB resistance and yield performance of 30 maize genotypes at two contrasting hotspot locations during the 2022–2023 seasons, to assess seasonal and spatial variation in resistance and yield traits; evaluate evidence for resistance breakdown in obsolete varieties (BH140, BH540) and the banned hybrid BH543; validate BH543 as a susceptible check; analyze disease progression patterns and perform mean separations per hotspot; compare hybrids with female parents for heterosis in resistance and yield; examine disease‐yield correlations; deliver location‐specific variety recommendations for different hotspot types; and identify elite resistance donors for sustainable maize breeding programs targeting Ethiopia's NLB hotspots (Haile et al. 2026a; Haile Onu et al. 2026).

## 2 Materials and Methods

### 2.1 Experimental Sites and Design

Field trials were conducted during the 2022 and 2023 main cropping seasons at two NLB hotspot locations in Ethiopia: Arsi Negele (7°20′ N, 38°38′ E; 1960 m above sea level) in the West Arsi Zone of Oromia Region, and Wondo Genet (6°54′ N, 38°38′ E; 1720 m above sea level) in the Sidama Region. These locations represent contrasting agro‐ecologies: Arsi Negele is a transitional highland with cooler temperatures and moderate rainfall, while Wondo Genet is a warm, humid mid‐altitude zone with higher disease pressure (Terefe et al. 2023; Haile et al. 2026a). Planting was done at the onset of the main rainy season (June at both locations in both years), consistent with recommendations for NLB hotspot management (Haile et al. 2026a).

Thirty maize genotypes were evaluated, comprising 24 commercial hybrids released between 1988 and 2020 and six female parent inbred lines (Table 1). The panel included obsolete varieties (BH140, BH540), the banned susceptible check BH543, widely cultivated hybrids (BH661, BH546, BH549, BH520), and their corresponding female parents. The experimental design was a randomized complete block design (RCBD) with three replications at each location. Each plot consisted of four rows 5 m long, with 0.75 m between rows and 0.25 m between plants. The two central rows constituted the net plot for data collection, while the outer rows served as borders.

**TABLE 1:** Maize genotypes evaluated for NLB resistance and yield performance.

No.	Genotype	Release year	Type	Source
1	BH140	1988	Hybrid	EIAR‐BRC
2	BH540	1995	Hybrid	EIAR‐BRC
3	BH660	2000	Hybrid	EIAR‐BRC
4	BH543	2005	Hybrid	EIAR‐BRC
5	BH661	2011	Hybrid	EIAR‐BRC
6	P30G19	2006	Hybrid	Corteva
7	P2859W	2011	Hybrid	Corteva
8	AMH850	2008	Hybrid	EIAR‐ARC
9	AMH851	2009	Hybrid	EIAR‐ARC
10	BH520	2020	Hybrid	EIAR‐BRC
11	BH546	2013	Hybrid	EIAR‐BRC
12	BH547	2013	Hybrid	EIAR‐BRC
13	BH549	2017	Hybrid	EIAR‐BRC
14	DK777	2017	Hybrid	Bayer
15	MH138Q	2012	Hybrid	EIAR‐MRC
16	MH140	2013	Hybrid	EIAR‐MRC
17	MH141	2020	Hybrid	EIAR‐MRC
18	P2809W	2017	Hybrid	Corteva
19	P3506W	2015	Hybrid	Corteva
20	P3812W	2012	Hybrid	Corteva
21	AMHQ‐760	2014	Hybrid	EIAR‐ARC
22	SBRH	2015	Hybrid	EIAR‐BRC
23	SPRH	2015	Hybrid	EIAR‐BRC
24	BHQY545	2008	Hybrid	EIAR‐BRC
25	F‐BH548	—	Parent of BH548	EIAR‐BRC
26	F‐BH661	—	Parent of BH661 and BH546	EIAR‐BRC
27	F‐BH660	—	Parent of BH660	EIAR‐BRC
28	F‐BH547	—	Parent of BH547	EIAR‐BRC
29	F‐BH543	—	Parent of BH543	EIAR‐BRC
30	F‐MH141	—	Parent of MH141	EIAR‐BRC

*Note:* EIAR‐BRC, EIAR‐ARC, and EIAR‐MRC represent Bako, Ambo, and Melkasa Agricultural Research Centers of the Ethiopian Institute of Agricultural Research, respectively.

### 2.2 Inoculum Preparation and Pathogen Confirmation

The use of ground infected leaf tissue as inoculum was chosen to preserve the natural pathogen population diversity present in Ethiopian hotspots, providing a more realistic resistance screening than single‐isolate inoculation while mimicking natural infection conditions where inoculum is dispersed from infected crop residues (Chandrashekara et al. 2014; Jindal et al. 2019; Haile et al. 2026a). Infected leaf tissue showing typical NLB symptoms (characteristic gray‐green to tan elliptical lesions with water‐soaked margins, measuring 2–15 cm in length) was collected from Wondo Genet research station during the peak disease period. To minimize contamination, leaves showing symptoms of other foliar diseases (rust pustules, streak virus symptoms, gray leaf spot lesions, or curvularia leaf spot) were strictly excluded. All collected tissue was inspected by two trained plant pathologists, and only tissue with more than 90% NLB lesions was retained. The collected tissue underwent a two‐step surface sterilization procedure: a 30‐s immersion in 70% ethanol followed by a 2‐min immersion in 1% sodium hypochlorite solution, then three rinses in sterile distilled water (Jindal et al. 2019). After surface sterilization, the tissue was air‐dried under sterile conditions in a laminar flow hood at 25°C for 48 h before being ground into a fine powder using a laboratory sample grinder mill (Retsch SM 100, Germany) equipped with a 2 mm mesh screen.

Pathogen identity was confirmed for each inoculum batch through microscopic examination of conidial morphology, with *E. turcicum* conidia characteristically appearing ellipsoid to fusiform, darkly pigmented (olivaceous‐brown), with 1–6 pseudosepta, measuring 80–130 × 20–30 μm (Leonard and Suggs 1974). A minimum of 50 conidia per batch were examined, and identity was confirmed when more than 95% of observed conidia matched these characteristics. Additionally, cultural confirmation was performed by plating representative samples onto potato dextrose agar (PDA) acidified to pH 4.5 with lactic acid and amended with streptomycin sulfate (100 mg L^−1^), incubated at 25°C under alternating 12‐h light/dark cycles for 7–10 days. *E. turcicum* colonies produce characteristic olivaceous‐gray to dark gray mycelium with a velvety to felt‐like texture, and sporulation was confirmed by microscopic examination of the colonies.

### 2.3 Inoculum Viability Testing and Quantification

Inoculum viability was tested for each batch prior to field inoculation using serial dilution plating. One gram of ground infected leaf tissue was suspended in 10 mL of sterile distilled water containing 0.1% Tween 20, vortexed for 2 min, and allowed to settle for 5 min. Ten‐fold serial dilutions (10^−1^ through 10^−6^) were prepared, and 100 μL of each dilution was plated onto PDA amended with streptomycin sulfate (100 mg L^−1^). Plates were incubated at 25°C for 7 days, and colony‐forming units (CFU) were counted on plates yielding 30–300 colonies. Only inoculum batches with a minimum viable concentration of 1 × 10^4^ CFU g^−1^ (fresh weight basis) were used for field inoculation. Viability testing was performed 48 h prior to each scheduled inoculation date, and inoculum was stored at 4°C in sealed containers until use. In practice, batch viabilities ranged from 1.2 × 10^4^ to 8.7 × 10^4^ CFU g^−1^ across the four environment combinations. The ground material was mixed with sterile talc powder (1:1 ratio, *w*/*w*) as a carrier, and the final concentration was adjusted to approximately 1 × 10^4^ CFU g^−1^ of the mixture. For each plant within the two central rows of every plot, approximately 0.2 g of the prepared inoculum mixture was applied, equivalent to approximately 2 × 10^3^ CFU per plant, a rate determined from preliminary trials to achieve greater than 50% disease severity on the susceptible check BH543 by physiological maturity.

### 2.4 Inoculation Procedure and Environmental Monitoring

Inoculations were performed using two complementary approaches to ensure comprehensive and uniform infection. Approximately 0.2 g of the prepared inoculum mixture was placed directly into the leaf whorl of each plant within the two central rows of every plot (Chandrashekara et al. 2014). To supplement the whorl application and ensure even distribution across the canopy, a fine dusting of the same inoculum mixture was applied over the canopy using a hand‐held bellows duster during periods of high humidity. Two separate inoculations were performed at critical growth stages: the first at the V6–V8 leaf stage (approximately 40–50 days after planting) and the second at the V10–V12 leaf stage (approximately 55–65 days after planting), just before canopy closure.

Environmental conditions during inoculation were monitored using a portable weather station (HOBO U30, Onset Computer Corporation, USA) that recorded air temperature, relative humidity, leaf wetness duration, rainfall, and wind speed at 15‐min intervals throughout the inoculation period and for 72 h post‐inoculation. Inoculation proceeded only when conditions met specific criteria: temperature between 18°C and 28°C, relative humidity exceeding 80%, forecasted leaf wetness duration of at least 12 h post‐inoculation, and wind speed less than 3 m s^−1^ (Magarey et al. 2005). When natural conditions were suboptimal for leaf wetness duration, supplemental overhead irrigation (5 mm) was applied immediately after inoculation and again 6 h later. Environmental conditions were consistently favorable across all environments: at Arsi Negele, temperatures averaged 21.3°C–22.1°C with relative humidity of 84.5%–86.2% and leaf wetness duration of 14.5–16.2 h; at Wondo Genet, temperatures averaged 22.8°C–23.4°C with relative humidity of 88.3%–89.7% and leaf wetness duration of 17.8–18.5 h.

### 2.5 Uniformity Assessment and Disease Evaluation

To ensure uniform disease pressure across all experimental units, four rows of the highly susceptible hybrid BH543 were planted around the border of the entire experiment and after every ten experimental plots as spreader rows, inoculated using the same protocol to serve as a continuous source of secondary inoculum. Uniformity was quantitatively assessed by calculating the coefficient of variation (CV) for NLB severity across spreader rows at the NLB6 stage, with an acceptance threshold of CV less than 15% (Megersa et al. 2017). Actual CV values ranged from 8.5% to 12.3% across the four environment combinations, confirming adequate uniformity. The randomized complete block design, with replications arranged perpendicular to the prevailing wind direction, further ensured that any spatial variation was accounted for in statistical analyses.

Disease severity was assessed six times at approximately 10‐day intervals from 60 to 110 days after planting, with the final assessment (NLB6) recorded at the dough stage (R4), using a modified 1–9 scale: 1 = no symptoms; 2 = ≤ 5% leaf area affected; 3 = 6%–10%; 4 = 11%–20%; 5 = 21%–30%; 6 = 31%–40%; 7 = 41%–50%; 8 = 51%–75%; 9 = > 75% (CIMMYT 1994). All disease ratings were conducted by the same two trained evaluators throughout the study to minimize scorer bias, with the inter‐evaluator correlation being high (*r* = 0.92, *p* < 0.001). Every 10th plot was reassessed by both evaluators as a quality control check. During assessment, plants showing symptoms of other foliar diseases (rust, streak virus, gray leaf spot) were excluded from NLB assessments; in practice, such plants represented less than 2% of the total and were consistent across genotypes. The six sequential ratings (NLB1–NLB6) were used to derive the following quantitative disease parameters:

#### 2.5.1 Percent Severity Index (PSI)

The ordinal severity scores were converted to a continuous percentage scale following McKinney (1923):

$$\mathrm{PSI}=\frac{\sum_{i=n}^{k}\left(S_{i}\times F_{i}\right)}{N\times S_{\max}}\times 100$$

where *S*_*i*_ is the severity score of the *i*‐th class, *F*_*i*_ is the frequency of plants in that score class, *k* is the number of score classes, *N* is the total number of plants assessed per plot, and *S*_max_ is the maximum score on the rating scale (9). PSI was calculated for each plot at each assessment date, with the final assessment designated as NLB6.

#### 2.5.2 Area Under Disease Progress Curve (AUDPC)

AUDPC was computed from the six sequential PSI values using the trapezoidal integration method (Shaner and Finney 1977) to integrate disease intensity over time:

$$\text{AUDPC}=\sum_{i=1}^{n-1}\left(\frac{y_{i+}y_{i+1}}{2}\;\right)\times \left(t_{i+1-}t_{i}\right)$$

where *y*_*i*_ is the PSI (%) at the *i*‐th assessment, *t*_*i*_ is the time in days after planting at the *i*‐th assessment, and *n* is the total number of assessments (6). AUDPC values are expressed in percent‐days.

#### 2.5.3 Apparent Infection Rate (*r*)

The apparent infection rate was calculated using the logistic transformation of disease progress (Plank 1963):

$$r=\frac{1}{t_{n}-t_{1}}\left[\ln\left(\frac{y_{n}}{1-y_{n}}\right)-\ln\left(\frac{y_{1}}{1-y_{1}}\right)\right]$$

where *y*_1_ and *y*_*n*_ are disease severities at the first and final assessments expressed as proportions (PSI/100), and *t*_1_ and *t*_*n*_ are the corresponding days after planting. Higher *r* values indicate more rapid epidemic development.

#### 2.5.4 Lesion Size (LS)

Lesion size was measured at the time of peak disease severity (NLB4 or NLB5, depending on genotype and environment). Ten representative lesions were randomly selected from the middle canopy leaves (leaf positions 6–10 above the ear) of each plot. Lesion length (*L*, mm) and width (*W*, mm) were measured using a digital caliper (0.01 mm precision). Lesion area was calculated assuming an elliptical shape (Carpane et al. 2020):

$$\mathrm{LS}=\pi \times \left(\frac{L}{2}\right)\times \left(\frac{W}{2}\right)\times \left(\frac{1}{100}\right)$$

where LS is expressed in cm^2^.

### 2.6 Yield Assessment

At physiological maturity, ears from the two central rows were hand‐harvested, shelled, and dried to 12.5% moisture content. Grain yield (GY) was calculated per hectare based on net plot area.

### 2.7 Statistical Analysis

All data were entered into Microsoft Excel spreadsheets and checked for errors and outliers. Descriptive statistics (means, standard deviations, ranges) were computed for each trait by location and year. Normality assumptions were tested using the Shapiro–Wilk test, and homogeneity of variance was assessed using Levene's test. Data from both years were first analyzed separately to test homogeneity of error mean squares before conducting combined analysis.

Data were combined for analysis using R version 4.5.2 (R Core Team 2024). Mixed model analysis was performed using the **lmer** function from the **lme4** package (Bates et al. 2015). The full linear mixed model was specified as:

$$\begin{array}{c} Y_{\text{ijkl}\;=\;}\mu +G_{i}+Y_{j}+L_{k}+{\left(G\times Y\right)}_{ij}+{\left(G\times L\right)}_{ik}+{\left(Y\times L\right)}_{ij}\\ \;+{\left(G\times Y\times L\right)}_{ijk}+R{\left(Y\times L\right)}_{i\left(j,k\right)}+\varepsilon_{\text{ijkl}} \end{array}$$

where *Y* is the observed trait value; *μ* is the overall mean; *G* is the fixed effect of the *i*‐th genotype; *Y* is the fixed effect of the *j*‐th year; *L* is the fixed effect of the *k*‐th location; *G* × *Y*, *G* × *L*, *Y* × *L*, and *G* × *Y* × *L* are fixed interaction effects; *R*(*Y* × *L*) is the random effect of the *l*th replication nested within year–location combinations; and *ε* is the residual error. Genotype, year, and location were treated as fixed effects because the 30 specific named commercial hybrids and the two deliberately chosen years and contrasting hotspot locations were selected for direct inference rather than random sampling from larger populations. Residual diagnostics included examination of *Q*–*Q* plots and residuals versus fitted values to verify normality and homoscedasticity. Percentage data (NLB severity, AUDPC) were arcsine‐square‐root transformed prior to analysis to stabilize variance; back‐transformed means are presented.

Type III analysis of variance with Satterthwaite's approximation for denominator degrees of freedom was performed using the **lmerTest** package. Due to significant genotype × location interactions (*p* < 0.001), separate ANOVAs were performed per location. Significant genotype effects (*p* < 0.05) were followed by Tukey's Honestly Significant Difference test (*α* = 0.05) using the **agricolae** package. Standard errors (SE) of means were calculated from the residual mean square and reported for all treatment means.

Repeated disease severity measures (NLB1–NLB6) were used to calculate AUDPC and apparent infection rate; ANOVA was performed on the derived summary variables (NLB6, AUDPC, *r*, LS, GY) rather than on longitudinal scores directly.

### 2.8 Heterosis Analysis

Female‐parent relative heterosis was calculated for selected hybrid–female parent pairs, with particular focus on the F‐BH661 family (BH661 and BH546), following Hallauer et al. (2010):

$$\begin{array}{c} \text{Female Parent Relative Heterosis}\;\left(\%\right)\\ =\left(\frac{\text{Hybrid performance}-\text{Female parent performance}}{\text{Female parent performance}}\right)\times 100 \end{array}$$

because male parents were not included in this study, this metric estimates the hybrid's deviation from the seed parent rather than true mid‐parent heterosis. For final NLB severity (NLB6), negative heterosis values indicate improved resistance (lower disease severity) in the hybrid compared to the female parent, while positive values indicate reduced resistance (increased susceptibility). For grain yield, positive heterosis values indicate yield improvement in the hybrid over the female parent.

## 3 Results

### 3.1 Efficacy of Inoculation and Disease Development

The artificial inoculation protocol resulted in uniform and severe NLB development across both hotspots and both years. First symptoms appeared 10–14 days after the second inoculation (approximately 55–60 days after planting), with rapid progression throughout grain‐filling (Figure 1). Disease development was more rapid and severe at Wondo Genet (mid‐altitude hotspot) due to more favorable environmental conditions (higher humidity, warmer temperatures), while Arsi Negele (transitional highland) showed moderate but adequate pressure for effective genotypic discrimination.

**FIGURE 1:**
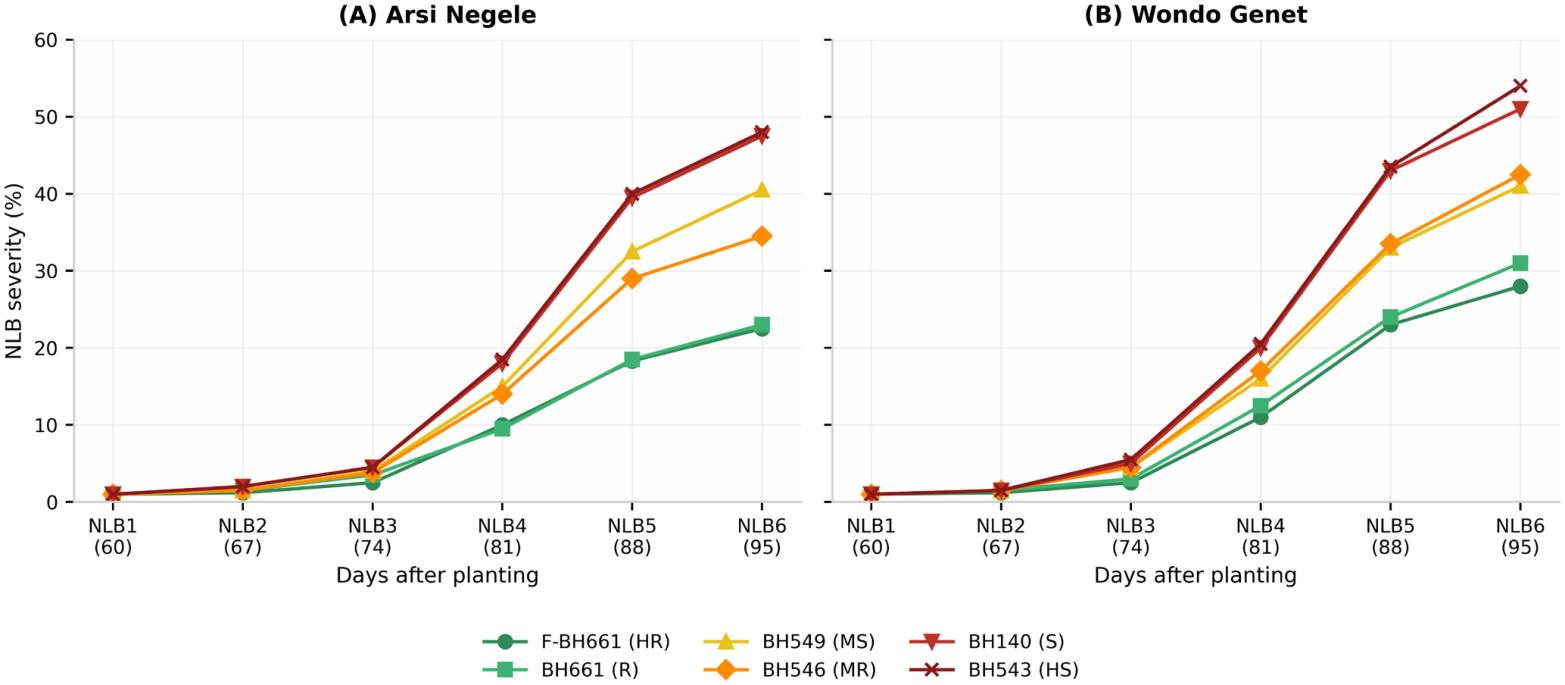
Northern leaf blight disease progress curves (NLB1–NLB6) for six representative maize genotypes at Arsi Negele and Wondo Genet (2022–2023). Solid lines represent fitted logistic curves; symbols indicate observed severity scores. HR = highly resistant (F‐BH661); HS = highly susceptible (BH543); MR = moderately resistant (BH546); MS = moderately susceptible (BH549); R = resistant (BH661); S = susceptible (BH140).

### 3.2 Summary Statistics and Environmental Characterization

Considerable variation was observed among genotypes for all traits, with notable differences between locations and years (Table 2). Mean NLB6 severity was consistently higher at Wondo Genet (50.7%–50.8%) compared to Arsi Negele (40.3%–45.5%), confirming higher disease pressure at the mid‐altitude hotspot. Grain yield was higher at Arsi Negele (5.51–5.77 t ha^−1^) than at Wondo Genet (4.64–5.37 t ha^−1^), reflecting both lower disease pressure and more favorable growing conditions at the transitional highland site.

**TABLE 2:** Summary statistics (mean ± standard deviation) for disease and yield traits across NLB hotspots.

Location	Year	NLB6 (%)	AUDPC (%—days)	*r* (day^−1^)	LS (cm^2^)	GY (t ha^−1^)
Arsi Negele	2022	40.3 ± 14.1	931.6 ± 326.5	0.054 ± 0.016	27.6 ± 11.8	5.77 ± 0.95
Arsi Negele	2023	45.5 ± 18.3	1070.6 ± 449.3	0.058 ± 0.021	29.9 ± 14.8	5.51 ± 0.82
Wondo Genet	2022	50.8 ± 15.9	1142.5 ± 407.3	0.065 ± 0.020	31.6 ± 12.6	5.37 ± 0.86
Wondo Genet	2023	50.7 ± 20.8	1218.6 ± 496.4	0.059 ± 0.029	29.6 ± 12.9	4.64 ± 0.84

*Note:* Values are means ± standard deviation (*n* = 90 plot observations per location–year combination: 30 genotypes × 3 replications).

Abbreviations: AUDPC, area under disease progress curve; GY, grain yield; LS, lesion size; NLB6, final northern leaf blight severity; *r*, apparent infection rate.

### 3.3 Analysis of Variance

The combined ANOVA revealed significant effects of genotype, year, location, and their interactions for most traits (Table 3). Genotype effects were highly significant (*p* < 0.001) for all traits. Critically, genotype × location interactions were highly significant for all traits (*p* < 0.001), demonstrating that genotype rankings differed between the two hotspot types and justifying location‐specific analyses and recommendations.

**TABLE 3:** Combined analysis of variance for disease and yield traits across NLB hotspots (*F*‐values and *p*‐values for all sources of variation).

Source of variation	df	NLB6	AUDPC	*r*	LS	GY
		*F*/*p*	*F*/*p*	*F*/*p*	*F*/*p*	*F*/*p*
Genotype (*G*)	29	12.4/< 0.001	9.8/< 0.001	8.5/< 0.001	6.2/< 0.001	15.3/< 0.001
Year (*Y*)	1	18.6/< 0.001	14.2/< 0.001	3.4/0.068	0.03/0.863	22.1/< 0.001
Location (*L*)	1	45.2/< 0.001	38.5/< 0.001	12.8/< 0.001	5.6/0.021	31.7/< 0.001
*G* × *Y*	29	3.2/< 0.001	2.8/< 0.001	2.5/< 0.001	2.1/0.012	4.1/< 0.001
*G* × *L*	29	4.5/< 0.001	3.9/< 0.001	3.2/< 0.001	4.8/< 0.001	5.6/< 0.001
*Y* × *L*	1	15.3/< 0.001	11.2/< 0.001	8.9/< 0.001	8.1/0.005	28.4/< 0.001
*G* × *Y* × *L*	29	2.1/< 0.001	1.9/< 0.001	1.8/< 0.001	1.4/0.054	3.2/< 0.001

*Note:* Type III *F*‐tests with Satterthwaite's approximation for denominator degrees of freedom.

Abbreviations: AUDPC, area under disease progress curve; G, genotype; GY, grain yield; L, location; LS, lesion size; NLB6, final NLB severity; *r*, apparent infection rate; Y, year.

### 3.4 Susceptibility of Obsolete Varieties Under Current Hotspot Conditions

Obsolete varieties BH140 (released 1988) and BH540 (released 1995) exhibited high NLB6 values (48.7% and 48.8%, respectively), statistically similar to the banned susceptible check BH543 (51.3%) (Table 4). At Wondo Genet, BH140 reached 50.3% severity and BH540 reached 50.0%, approaching BH543's 53.8%, while at Arsi Negele both showed 47.0%–47.5% severity. This susceptibility is nearly double that of resistant hybrids such as BH661 (26.8% NLB6). Whether this reflects resistance breakdown due to pathogen evolution, inherently moderate original resistance, or environmental maladaptation cannot be determined from the present data and requires historical resistance records and pathogen race characterization for confirmation. BH543 exhibited the highest overall NLB6 (51.3%), with particularly severe disease at Wondo Genet (53.8%), confirming its appropriateness as a susceptible check.

**TABLE 4:** Performance of obsolete varieties compared to resistant and susceptible checks (mean ± SE).

Genotype	Category	Final NLB severity (%)			Mean GY (t ha^−1^)
		Arsi Negele	Wondo Genet	Overall	
BH140	Obsolete (1988)	47.0 ± 2.1ᵃ	50.3 ± 2.4ᵃ	48.7 ± 1.8ᵃ	5.33 ± 0.22
BH540	Obsolete (1995)	47.5 ± 2.3ᵃ	50.0 ± 2.6ᵃ	48.8 ± 1.9ᵃ	5.18 ± 0.24
BH543	Banned (2005)	48.7 ± 2.5ᵃ	53.8 ± 2.8ᵃ	51.3 ± 2.1ᵃ	4.66 ± 0.26
F‐BH661	Resistant parent	22.5 ± 2.0ᶜ	27.8 ± 2.3ᶜ	25.1 ± 1.5ᶜ	5.84 ± 0.18
BH661	Resistant hybrid	23.5 ± 2.1ᶜ	30.0 ± 2.4ᶜ	26.8 ± 1.6ᶜ	5.87 ± 0.18
BH549	High‐yield	40.5 ± 2.0ᵇ	40.5 ± 2.2ᵇ	40.5 ± 1.7ᵇ	6.80 ± 0.25

*Note:* Values followed by different superscript letters within the Overall column differ significantly at *p* < 0.05 (Tukey's HSD). Letter groupings apply within columns.

Abbreviations: GY, grain yield; NLB, northern leaf blight.

### 3.5 F‐BH661 as an Elite Resistance Source

F‐BH661 exhibited the lowest mean NLB6 severity (25.1%) among all 30 genotypes, maintaining exceptional resistance across both hotspot types (22.5% at Arsi Negele, 27.8% at Wondo Genet) with a mean yield of 5.84 t ha^−1^ (Table 5). As the female parent of both BH661 (2011) and BH546 (2013), F‐BH661 has contributed resistance to two commercial hybrids, but the contrasting performance of its derived hybrids provides critical insights into resistance inheritance and male parent effects.

**TABLE 5:** Performance of F‐BH661 and its derived hybrids BH661 and BH546 (mean ± SE).

Genotype	Type	Final NLB severity (%)			Grain yield (t ha^−1^)		
		AN	WG	Mean	AN	WG	Mean
F‐BH661	Female parent	22.5 ± 2.0	27.8 ± 2.3	25.1 ± 1.6ᶜ	6.10 ± 0.22	5.58 ± 0.24	5.84 ± 0.18
BH661	Hybrid	23.5 ± 2.1	30.0 ± 2.4	26.8 ± 1.6ᶜ	6.25 ± 0.23	5.49 ± 0.24	5.87 ± 0.18
BH546	Hybrid	35.5 ± 2.4	41.5 ± 2.7	38.5 ± 2.0ᵃᵇ	6.40 ± 0.24	5.20 ± 0.25	5.80 ± 0.19

*Note:* Standard errors were calculated as √(EMS/*n*), where EMS is the error mean square from the location‐specific ANOVA and *n* = 6 (3 replications × 2 years) for location‐specific means and *n* = 12 (3 replications × 2 years × 2 locations) for overall means. Values followed by different superscript letters within the NLB6 Mean column differ significantly at *p* < 0.05 (Tukey's HSD).

Abbreviations: AN, Arsi Negele; SE, standard error of the mean; WG, Wondo Genet.

### 3.6 Female‐Parent Relative Heterosis for NLB Resistance and Grain Yield

Heterosis values for NLB6 ranged from +6.7% to +53.4% across hybrid–female parent pairs (Table 6), demonstrating that the same female parent can produce hybrids with dramatically different resistance outcomes depending on the male parent. BH661 demonstrated slight positive heterosis for NLB6 (+6.7%), indicating a marginal increase in susceptibility relative to F‐BH661, though the hybrid retained high overall resistance statistically similar to the female parent. This suggests that the male parent (142‐1‐e) did not contribute additional resistance alleles at the major loci underlying F‐BH661's elite performance. In contrast, BH546 showed positive heterosis (+53.4%), indicating that the male parent (BKL001) contributed susceptibility alleles that substantially eroded F‐BH661 resistance.

**TABLE 6:** Female‐parent relative heterosis for grain yield and NLB resistance in hybrid–female parent pairs.

Genotype		GY (t/ha)			NLB6 (%)		
Hybrid	Female	Hybrid	Female	Heterosis (%)	Hybrid	Female	Heterosis (%)
BH661	F‐BH661	5.87	5.84	+0.5	26.8	25.1	+6.7
BH546	F‐BH661	5.80	5.84	−0.7	38.5	25.1	+53.4
BH660	F‐BH660	4.81	4.38	+9.8	31.8	33.0	−3.6
BH547	F‐BH547	4.56	4.58	−0.4	50.2	48.5	+3.5
BH543	F‐BH543	4.66	4.42	+5.4	51.3	53.5	−4.1
MH141	F‐MH141	5.36	4.89	+9.6	42.5	44.8	−5.1

*Note:* Negative heterosis for final NLB severity (NLB6) indicates improved resistance (lower disease severity) in the hybrid compared to the female parent. Because male parents were not evaluated in this study, this metric represents female‐parent relative heterosis rather than true mid‐parent heterosis.

Abbreviation: GY, grain yield.

### 3.7 Classification of Commercial Hybrids

The 30 genotypes were classified into six resistance categories based on mean NLB6 severity (Figure 2). Only three genotypes—F‐BH661 (25.1%), BH661 (26.8%), and AMH851 (29.3%)—possessed exceptional resistance (≤ 30% NLB6) suitable for high‐pressure environments. Eight genotypes were moderately resistant (35.1%–40.0%), including DK777 (36.8%, 6.49 t ha^−1^) and BH546 (38.5%, 5.80 t ha^−1^). High‐yielding hybrids BH549 (40.5%, 6.80 t ha^−1^) and BH520 (40.5%, 6.64 t ha^−1^) were moderately susceptible.

**FIGURE 2:**
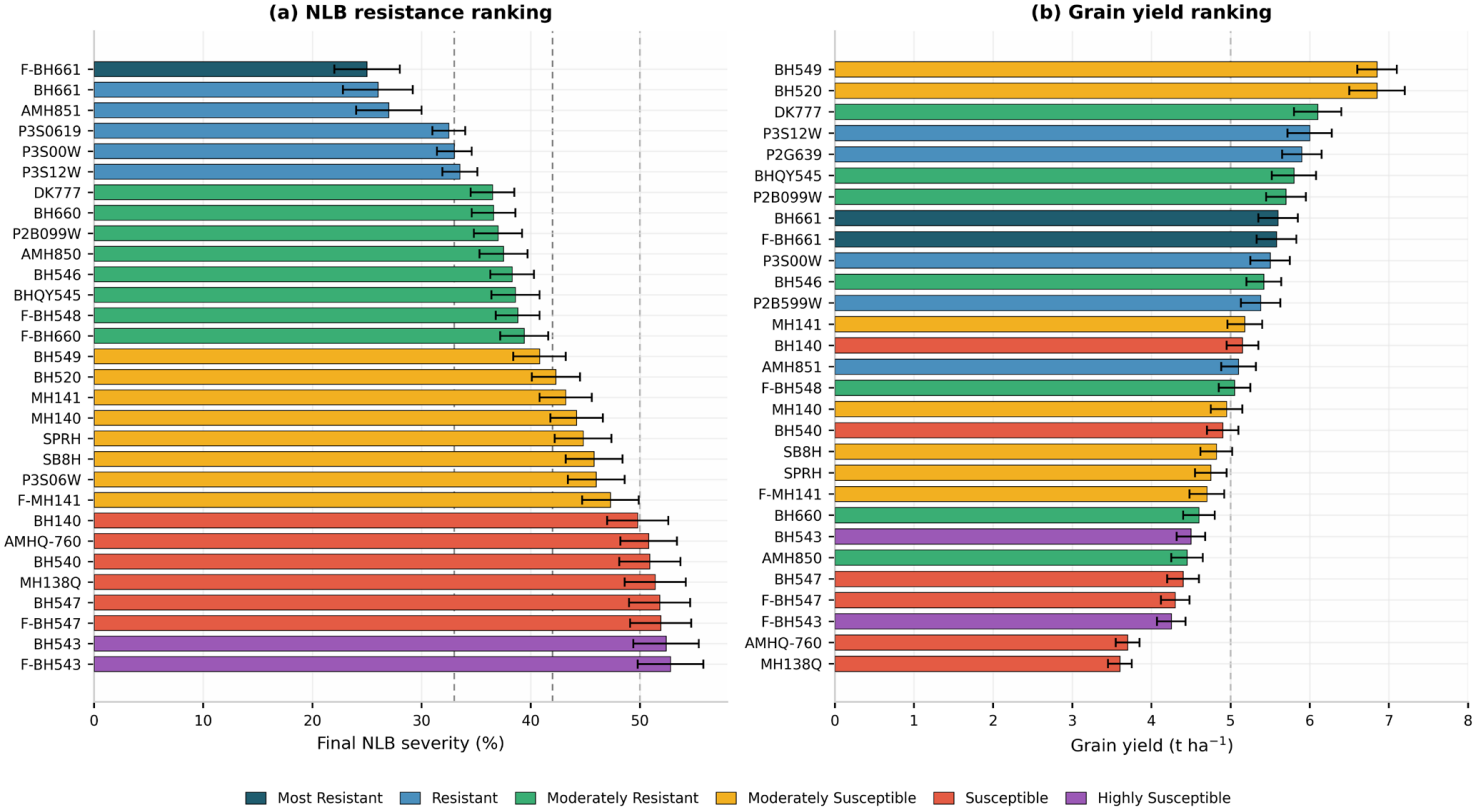
Genotype ranking for final NLB severity and grain yield across two NLB hotspots. Error bars represent standard errors of the mean. Colors indicate resistance classification (Most Resistant to Highly Susceptible).

The *G* × *E* biplot (Figure 3) confirmed that genotype rankings differed between locations, with BH661 and F‐BH661 consistently projecting toward lower NLB severity in all environments, while BH549 and BH520 projected toward higher grain yield particularly at Arsi Negele.

**FIGURE 3:**
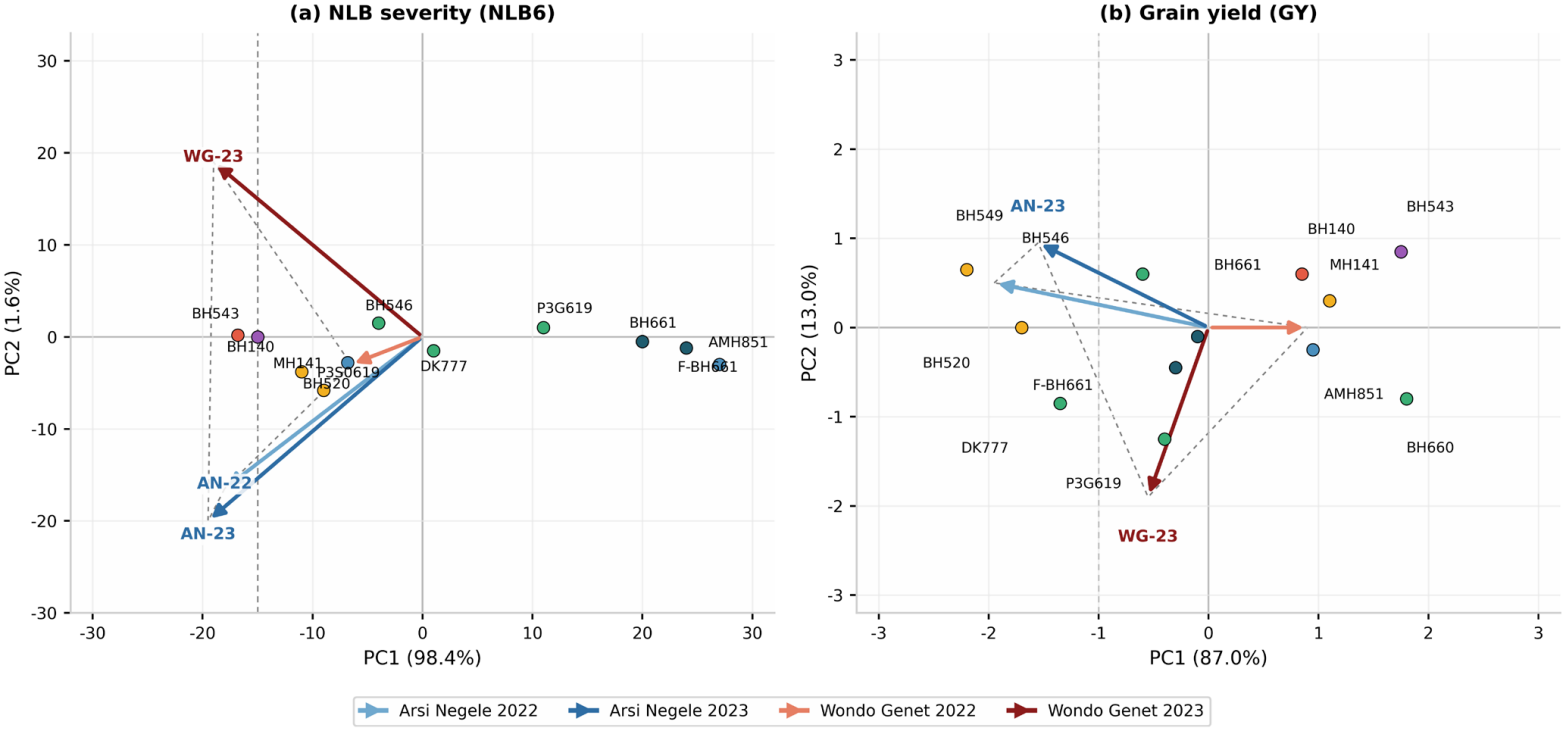
Genotype × environment biplots for NLB severity and grain yield across four environment combinations (Arsi Negele and Wondo Genet, 2022–2023), showing which‐won‐where patterns and mega‐environment differentiation.

### 3.8 Correlation Analysis

Very strong positive correlations were observed between NLB6 and AUDPC (*r* = 0.78**) and between NLB6 and infection rate (*r* = 0.90**), indicating that final disease severity is an excellent proxy for both cumulative disease experience and epidemic development rate. NLB6 showed a strong positive correlation with lesion size (*r* = 0.62**). A moderate negative correlation was observed between grain yield and NLB6 (*r* = −0.40**), confirming that NLB contributes to yield loss in hotspot areas, although yield is also influenced by multiple confounding factors including adaptation, maturity, environmental interactions, and genetic background beyond NLB severity alone (Table 7).

**TABLE 7:** Pearson correlation coefficients among key traits.

Trait	AUDPC	*r*	LS	GY	NLB6
AUDPC	1				
*r*	0.69**	1			
LS	0.53**	0.50**	1		
GY	−0.24*	−0.25*	−0.21*	1	
NLB6	0.78**	0.90**	0.62**	−0.40**	1

*Note:* Significant at *p* < 0.05 (*) and *p* < 0.01 (**), respectively. Correlation coefficients interpreted following Cohen (1988): |*r*| < 0.3 = weak; 0.3 ≤ |*r*| < 0.5 = moderate; 0.5 ≤ |*r*| < 0.7 = strong; |*r*| ≥ 0.7 = very strong.

Abbreviations: AUDPC, area under disease progress curve; GY, grain yield; LS, lesion size; NLB6, final NLB severity; *r*, apparent infection rate.

## 4 Discussion

### 4.1 Susceptibility of Obsolete Varieties and Resistance Durability

The high susceptibility of obsolete varieties BH140 and BH540 under current hotspot conditions raises important questions about the durability of NLB resistance in Ethiopian maize germplasm. Large‐scale surveys confirm that BH540 suffers severe NLB infection (mean PSI 77.1%) under current Ethiopian growing conditions, whereas modern resistant hybrids such as BH661 maintain significantly lower severity (PSI 51.7%) (Haile et al. 2026a). Several non‐mutually exclusive hypotheses may explain this pattern. First, these varieties may have possessed effective resistance at release that has been overcome by pathogen evolution over 17–27 years of widespread cultivation. *E. turcicum* is known to exist in multiple physiological races with differential virulence profiles (Bankole et al. 2023; Welz and Geiger 2000), and selection pressure from major‐gene resistance can drive population shifts toward virulent races within 5–15 years (Michel et al. 2023). Second, BH140 and BH540 may have possessed only moderate quantitative resistance that was sufficient under lower historical disease pressure but is inadequate in contemporary high‐inoculum hotspots. Third, environmental maladaptation—given that these varieties were bred for mid‐altitude conditions that may have differed from current hotspot microclimates—could contribute to their poor performance. Without historical resistance data from controlled trials at release and contemporary pathogen race characterization, definitive conclusions regarding resistance breakdown cannot be drawn (Delmotte et al. 2016; McDonald and Linde 2002a). This uncertainty underscores the need for systematic pathogen surveys and sentinel plot monitoring in Ethiopian maize production zones.

### 4.2 F‐BH661 Resistance and Male Parent Effects

The identification of F‐BH661 as an elite resistance source (25.1% NLB6) with stable performance across contrasting hotspot types (22.5% at Arsi Negele, 27.8% at Wondo Genet) suggests that its resistance is quantitatively inherited and environmentally robust. This finding is consistent with field surveys identifying moist highland and semi‐moist mid‐altitude zones as high‐pressure environments where quantitative resistance is essential (Haile et al. 2026a). The contrasting performance of its half‐sib hybrids provides a compelling natural experiment in resistance genetics. BH661 maintained exceptional resistance (26.8% NLB6) with slight positive female‐parent relative heterosis (+6.7%), indicating a marginal increase in susceptibility relative to the female parent. The hybrid retained high overall resistance statistically similar to F‐BH661, suggesting that the male parent (142‐1‐e) neither enhanced nor substantially eroded the female parent's resistance at the major loci conferring elite performance. Conversely, BH546 showed dramatically reduced resistance (38.5% NLB6, +53.4% heterosis relative to F‐BH661), demonstrating that the male parent (BKL001) contributed susceptibility alleles that substantially eroded F‐BH661 resistance, underscoring the predominance of nonadditive gene action and specific combining ability in determining hybrid resistance levels (Haile et al. 2026b). The 11.7 percentage‐point difference in NLB6 between these half‐sib hybrids is entirely attributable to male parent effects. This contrast highlights a critical principle for hybrid breeding: even with an elite resistant female parent, male parent selection determines whether resistance is preserved, enhanced, or eroded in the commercial hybrid. Breeding programs should evaluate multiple male parent combinations with elite female lines and use the resistant hybrid BH661 as a benchmark (target ≤ 27% NLB6 under artificial inoculation). Multi‐environment diallel analyses further demonstrate that while general combining ability supports recurrent selection for resistance, the substantial dominance variance (62%–83% of genetic variance) for NLB severity and grain yield necessitates strategic hybrid exploitation through testcross evaluation (Haile et al. 2026b).

### 4.3 Genotype × Environment Interactions and Variety Recommendations

Significant *G* × *L* interactions (*p* < 0.001) for all traits confirm that no single variety is optimal across both hotspot types (Atlin et al. 2000). The *G* × *E* biplot revealed distinct which‐won‐where patterns: for NLB resistance, BH661 and F‐BH661 consistently outperformed all other genotypes in both environments, while for grain yield, BH549 and BH520 excelled primarily at Arsi Negele. This differentiation necessitates location‐specific recommendations. For mid‐altitude, high‐pressure hotspots (Wondo Genet type), where NLB6 means exceeded 50%, resistance must be the primary selection criterion—BH661 is the clear first choice, with P30G19, DK777, and P3812W as secondary options. Previous multi‐environment yield stability analyses similarly identified BH661, BH549, and BH546 as broadly adapted, high‐yielding genotypes suitable for variable conditions in Ethiopian maize production systems (Daemo et al. 2024).

For transitional highland, moderate‐pressure hotspots (Arsi Negele type), farmers can prioritize yield potential with moderate resistance: BH549 (6.80 t ha^−1^) and BH520 lead, with BH661 providing resistance insurance. Daemo et al. (2024) confirmed BH661 and BH549 as optimal choices for variable agro‐ecologies, with BH661 surpassing the national mean productivity by 130.9% and outperforming the standard check by 21.2%. Obsolete varieties BH140, BH540, and the banned hybrid BH543 should be avoided entirely in both environments, consistent with large‐scale epidemiological data classifying BH540 and local landraces among the most susceptible cultivars in Ethiopia (Haile et al. 2026a).

### 4.4 Correlations and Selection Criteria

The very strong correlation between NLB6 and infection rate (*r* = 0.90) indicates that final severity alone can serve as an effective proxy for epidemic development rate in screening programs, reducing the need for repeated assessments in early generations. However, the moderate correlation between NLB6 and grain yield (*r* = −0.40) suggests that while NLB significantly impacts productivity, selection for resistance alone will not maximize yield. Yield is also influenced by multiple confounding factors including adaptation, maturity, environmental interactions, and genetic background beyond NLB severity alone. This is consistent with diallel studies reporting a stronger yield–resistance correlation (*r* = −0.75) under controlled multi‐location trials, where dominance variance for both traits is substantial (Haile et al. 2026b). Simultaneous selection for both traits is necessary, particularly in moderate‐pressure environments where the yield–resistance trade‐off is most pronounced.

### 4.5 Implications for Breeding Durable Resistance

The sustained low severity of F‐BH661 and BH661 over the evaluation period, contrasted with the high susceptibility of obsolete varieties, suggests that the polygenic, partial resistance present in F‐BH661 may offer greater durability than major‐gene resistance that may have been present in earlier varieties. This pattern is consistent with the general principle that quantitative resistance, controlled by multiple genes with small individual effects, is more difficult for pathogen populations to overcome than qualitative, major‐gene resistance (Aubertot et al. 2006). However, confirming true durability requires genetic analysis (QTL mapping), knowledge of the resistance mechanisms, and long‐term monitoring across multiple pathogen generations. Breeding programs should therefore integrate recurrent selection to accumulate favorable resistance alleles with strategic hybrid deployment exploiting complementary gene action, as demonstrated by the transgressive performance of specific crosses in diallel evaluations (Haile et al. 2026b). We recommend that breeding programs prioritize: (i) recurrent selection to accumulate favorable resistance alleles; (ii) diversification of resistance sources to reduce selection pressure on pathogen populations; (iii) gene pyramiding where molecular markers are available; and (iv) continuous phenotypic selection under high disease pressure at both hotspot types.

## 5 Conclusions

This study evaluated 30 maize genotypes against NLB under artificial inoculation at two contrasting Ethiopian hotspots over two seasons, revealing significant genetic variation for resistance and yield with important implications for breeding and variety recommendations.

Obsolete varieties BH140 (48.7% NLB6) and BH540 (48.8% NLB6) exhibited high susceptibility under current hotspot conditions, statistically similar to the banned check BH543 (51.3%). Whether this reflects resistance breakdown due to pathogen evolution, inherently moderate historical resistance, or environmental maladaptation cannot be determined from the present data; pathogen race characterization and historical resistance records are required for confirmation. Field‐scale epidemiological surveys and fungicide efficacy trials underscore the severe yield penalty imposed by NLB in susceptible germplasm, with each 1% increase in R4 disease severity reducing seed yield by approximately 28 kg ha^−1^ (Haile Onu et al. 2026).

F‐BH661 was identified as an elite resistance source (25.1% NLB6) with stable resistance across both hotspot types and good yield (5.84 t ha^−1^), making it an ideal donor parent. Its derived hybrid BH661 is the most resistant commercial hybrid (26.8% NLB6, 5.87 t ha^−1^), with slight positive female‐parent relative heterosis (+6.7%), indicating a marginal increase in susceptibility relative to the female parent, though the hybrid retained high overall resistance statistically similar to F‐BH661. In contrast, BH546 shows significantly reduced resistance (38.5% NLB6, +53.4% heterosis relative to F‐BH661) due to male parental effects, highlighting the critical importance of male parent selection.

Significant genotype–location interactions (*p* < 0.001) necessitate hotspot‐specific recommendations: BH661 for mid‐altitude high‐pressure hotspots; BH549 and BH520 for transitional highland, moderate‐pressure areas. The 31.5% yield penalty of BH543 versus BH549 (4.66 vs. 6.80 t ha^−1^) validates the regulatory decision to ban highly susceptible germplasm.

Correlation analysis confirms NLB contributes to yield reduction (*r* = −0.40 with NLB6), while very strong correlations between NLB6 and both AUDPC (*r* = 0.78) and infection rate (*r* = 0.90) indicate final severity alone can serve as an effective selection criterion. Continuous resistance monitoring and breeding are essential, as the susceptibility of obsolete varieties demonstrates that resistance effectiveness is not permanent.

## Funding

No external funding was received for this study. The research was conducted as part of the core mandate of the Ethiopian Institute of Agricultural Research (EIAR).

## Conflicts of Interest

The authors declare no conflicts of interest. The authors alone are responsible for the content and writing of the manuscript.

## Supporting information


**Data S1:** Raw plot‐level data for the 30 maize genotypes evaluated in this study. The file contains disease severity scores (NLB1–NLB6), area under the disease progress curve (AUDPC), apparent infection rate (r), lesion size (LS), and grain yield (GY) for all plot observations recorded at two NLB hotspot locations (Arsi Negele and Wondo Genet) across two cropping seasons (2022 and 2023), with three replications per genotype per location–year combination.

## Data Availability

The data presented in this study are openly available in Data S1, the caption of which is provided in the Supporting Information section. Data S1 contains all plot‐level raw data (disease severity scores NLB1–NLB6, area under the disease progress curve [AUDPC], apparent infection rate [*r*], lesion size [LS], and grain yield [GY]) from the 30 genotypes evaluated across two locations (Arsi Negele and Wondo Genet) and two cropping seasons (2022 and 2023), with three replications per genotype per location. The authors confirm that this data availability statement, as stated in the manuscript, is correct and matches the file submitted with this manuscript. Additional data are available from the corresponding author upon reasonable request.

## References

[pei370219-bib-0001] Abate, T. , B. Shiferaw , A. Menkir , et al. 2015. “Factors That Transformed Maize Productivity in Ethiopia.” Food Security 7, no. 5: 965–981. 10.1007/s12571-015-0488-z.

[pei370219-bib-0003] Atlin, G. N. , R. J. Baker , K. B. McRae , and X. Lu . 2000. “Selection Response in Subdivided Target Regions.” Crop Science 40, no. 1: 7–13. 10.2135/cropsci2000.4017.

[pei370219-bib-0004] Aubertot, J. N. , J. S. West , L. Bousset‐Vaslin , M. U. Salam , M. J. Barbetti , and A. J. Diggle . 2006. “Improved Resistance Management for Durable Disease Control: A Case Study of Phoma Stem Canker of Oilseed Rape ( *Brassica napus* ).” European Journal of Plant Pathology 114, no. 1: 91–106. 10.1007/s10658-005-3628-z.

[pei370219-bib-0005] Bankole, F. A. , B. Badu‐Apraku , A. O. Salami , T. D. O. Falade , R. Bandyopadhyay , and A. Ortega‐Beltran . 2023. “Variation in the Morphology and Effector Profiles of *Exserohilum turcicum* Isolates Associated With the Northern Corn Leaf Blight of Maize in Nigeria.” BMC Plant Biology 23, no. 1: 386. 10.1186/s12870-023-04385-7. PMC10413532 37563555

[pei370219-bib-0006] Bates, D. , M. Mächler , B. M. Bolker , and S. C. Walker . 2015. “Fitting Linear Mixed‐Effects Models Using lme4.” Journal of Statistical Software 67, no. 1: 1–48. 10.18637/jss.v067.i01.

[pei370219-bib-0008] Carpane, P. D. , A. M. Peper , and F. Kohn . 2020. “Management of Northern Corn Leaf Blight Using Nativo (Trifloxistrobin + Tebuconazole) Fungicide Applications.” Crop Protection 127: 104982. 10.1016/j.cropro.2019.104982.

[pei370219-bib-0009] Chandrashekara, C. , S. K. Jha , R. Arunkumar , and P. K. Agrawal . 2014. “Identification of New Sources of Resistance to Turcicum Leaf Blight and Maydis Leaf Blight in Maize ( *Zea mays* L.).” Sabrao Journal of Breeding and Genetics 46, no. 1: 44–55.

[pei370219-bib-0010] Chivasa, W. , M. Worku , A. Teklewold , et al. 2022. “Maize Varietal Replacement in Eastern and Southern Africa: Bottlenecks, Drivers and Strategies for Improvement.” Global Food Security 32: 100589. 10.1016/j.gfs.2021.100589. PMC8907863 35300043

[pei370219-bib-0047] CIMMYT . 1994. Maize Diseases: A Guide for Field Identification. 4th ed. International Maize and Wheat Improvement Center (CIMMYT).

[pei370219-bib-0048] Cohen, J. 1988. Statistical Power Analysis for the Behavioral Sciences. 2nd ed. Lawrence Erlbaum Associates.

[pei370219-bib-0011] CSA . 2021. “The Federal Democratic Republic of Ethiopia Central Statistical Agency Report on Farm Management Practices.” Statistical Bulletin 3: 512.

[pei370219-bib-0012] Daemo, B. B. , G. B. Wolancho , Z. A. Arke , D. D. Wakalto , and M. H. Onu . 2024. “Performance Evaluation and Stability of Maize ( *Zea mays* L.) Genotypes for Grain Yield Using AMMI and GGE Biplot.” International Journal of Agronomy 2024: 8801999. 10.1155/2024/8801999.

[pei370219-bib-0013] Delmotte, F. , D. Bourguet , P. Franck , et al. 2016. “Combining Selective Pressures to Enhance the Durability of Disease Resistance Genes.” Frontiers in Plant Science 7: 1916. 10.3389/fpls.2016.01916. PMC5180194 28066472

[pei370219-bib-0014] Erenstein, O. , M. Jaleta , K. Sonder , K. Mottaleb , and B. M. Prasanna . 2022. “Global Maize Production, Consumption and Trade: Trends and R&D Implications.” Food Security 14, no. 5: 1295–1319. 10.1007/s12571-022-01288-7.

[pei370219-bib-0015] Gemechu, N. , T. Leta , A. Sentayehu , and W. Dagne . 2018. “Combining Ability of Selected Maize ( *Zea mays* L.) Inbred Lines for Major Diseases, Grain Yield and Selected Agronomic Traits Evaluated at Melko, South West Oromia Region, Ethiopia.” African Journal of Agricultural Research 13, no. 38: 1998–2005. 10.5897/ajar2018.13285.

[pei370219-bib-0016] Goshime, M. M. , T. M. Walter , A. S. Solomon , et al. 2021. “Identification of Best Maize‐Legume Based Cropping Systems Under Conservation Agriculture Practices for Central Rift Valley of Ethiopia.” Journal of Plant Breeding and Crop Science 13, no. 2: 35–45. 10.5897/jpbcs2020.0928.

[pei370219-bib-0017] Haile, M. , Z. Bekeko , D. Wegary , H. Terefe , and S. L. Mahabaleswara . 2026b. “Combining Ability and Gene Action for Northern Leaf Blight Resistance and Grain Yield in Tropical Maize ( *Zea mays* L.).” International Journal of Agronomy 2026: 1729008. 10.1155/2026/1729008.

[pei370219-bib-0046] Haile, M. , Z. Bekeko , D. Wegary , H. Terefe , and L. M. Suresh . 2026a. “Epidemics of Northern Leaf Blight (*Exserohilum turcicum*) as Influenced by Epidemiological Drivers and Agronomic Factors in the Major Maize‐Growing Agro‐Ecologies of Ethiopia.” Journal of Crop Health 78, no. 5. 10.1007/s10343-026-01394-y.

[pei370219-bib-0019] Haile Onu, M. , Z. Bekeko Erena , D. Wegary Gissa , H. Terefe Yitayew , and S. L. Mahabaleswara . 2026. “Efficacy of Dual‐Mode Fungicides in Management of Northern Leaf Blight (*Exserohilum turcicum*) and Quantification of Yield Losses in Ethiopian Seed Maize Systems.” Journal of Phytopathology 174: e70380.

[pei370219-bib-0020] Hallauer, A. R. , M. J. Carena , and J. B. Miranda Filho . 2010. Quantitative Genetics in Maize Breeding. Vol. 6. Springer Science and Business Media.

[pei370219-bib-0022] Jindal, K. K. , X. Zhu , T. Woldemariam , et al. 2019. “Maize Inbreds for Multiple Resistance Breeding Against Major Foliar, Ear and Stalk Rot Diseases.” Maydica 64, no. 1: 1–22.

[pei370219-bib-0023] Keno, T. , G. Azmach , D. Wegary , et al. 2018. “Major Biotic Maize Production Stresses in Ethiopia and Their Management Through Host Resistance.” African Journal of Agricultural Research 13, no. 21: 1042–1052. 10.5897/ajar2018.13163.

[pei370219-bib-0024] Leonard, K. J. , and E. G. Suggs . 1974. “ *Setosphaeria prolata*, the Ascigerous State of *Exserohilum prolatum* .” Mycologia 66, no. 2: 281–297.

[pei370219-bib-0025] Magarey, R. D. , R. C. Seem , A. Weiss , T. J. Gillespie , and L. Huber . 2005. “Estimating Surface Wetness on Plants.” In Micrometeorology in Agricultural Systems (Agronomy Monograph No. 47), edited by J. L. Hatfield , 199–226. American Society of Agronomy. 10.2134/agronmonogr47.c10.

[pei370219-bib-0026] Marenya, P. , R. Wanyama , S. Alemu , O. Westengen , and M. Jaleta . 2022. “Maize Variety Preferences Among Smallholder Farmers in Ethiopia: Implications for Demand‐Led Breeding and Seed Sector Development.” PLoS One 17, no. 9: e0274262. 10.1371/journal.pone.0274262. PMC9522265 36174004

[pei370219-bib-0027] McDonald, B. A. , and C. Linde . 2002a. “Pathogen Population Genetics, Evolutionary Potential, and Durable Resistance.” Annual Review of Phytopathology 40: 349–379. 10.1146/annurev.phyto.40.120501.101443. 12147764

[pei370219-bib-0028] McDonald, B. A. , and C. Linde . 2002b. “The Population Genetics of Plant Pathogens and Breeding Strategies for Durable Resistance.” Euphytica 124, no. 2: 163–180. 10.1023/A:1015678432355.

[pei370219-bib-0030] McKinney, H. H. 1923. “Influence of Soil Temperature and Moisture on Infection of Wheat Seedlings by *Helminthosporium sativum* .” Journal of Agricultural Research 26, no. 5: 195–217.

[pei370219-bib-0031] Megersa, D. , D. Mashilla , and A. Wende . 2017. “Management of Turcicum Leaf Blight [*Exserohilum turcicum* (Pass.) Leonard & Suggs] of Maize ( *Zea mays* L.) Through Integration of Host Resistance and Fungicide at Bako, Western Ethiopia.” African Journal of Plant Science 11, no. 1: 6–22. 10.5897/ajps2016.1449.

[pei370219-bib-0032] Michel, S. , F. Löschenberger , C. Ametz , and H. Bürstmayr . 2023. “Toward Combining Qualitative Race‐Specific and Quantitative Race‐Nonspecific Disease Resistance by Genomic Selection.” Theoretical and Applied Genetics 136, no. 4: 79. 10.1007/s00122-023-04312-2. PMC10036288 36952008

[pei370219-bib-0033] Ohunakin, A. O. , A. C. Odiyi , and L. S. Fayeun . 2023. “Genetic Analysis of Resistance to Northern Leaf Blight of Tropical Maize Inbred Lines.” Journal of Crop Science and Biotechnology 26, no. 5: 595–606. 10.1007/s12892.

[pei370219-bib-0034] Onwunali, O. O. , and R. B. Mabagala . 2020. “Northern Leaf Blight of Maize: A Review of the Disease, Pathogen and Host Resistance.” International Journal of Agronomy 2020: 8836338. 10.1155/2020/8836338.

[pei370219-bib-0035] Plank, J. E. 1963. Plant Diseases: Epidemics and Control. Academic Press.

[pei370219-bib-0049] R Core Team . 2024. R: A Language and Environment for Statistical Computing (Version 4.2) [Computer Software]. R Foundation for Statistical Computing. https://www.R‐project.org/.

[pei370219-bib-0036] Ramathani, I. , F. Beed , and A. Sparks . 2011. “Occurrence and Distribution of Maize Diseases in Smallholder Farms in the Mid and Low Altitude Zones of Rwanda.” Journal of Animal and Plant Sciences 11, no. 2: 1424–1434.

[pei370219-bib-0037] Shaner, G. , and R. E. Finney . 1977. “The Effect of Nitrogen Fertilization on the Expression of Slow‐Mildewing Resistance in Knox Wheat.” Phytopathology 67, no. 8: 1051–1056. 10.1094/Phyto-67-1051. 40934422

[pei370219-bib-0038] Tarekegne, A. , D. Makumbi , C. Magorokosho , J. E. Cairns , T. Ndhlela , and Y. Beyene . 2024. “Genetic Gains in Early Maturing Maize Hybrids Developed by the International Maize and Wheat Improvement Center in Southern Africa During 2000–2018.” Frontiers in Plant Science 15: 1321308. 10.3389/fpls.2023.1321308. PMC10825029 38293626

[pei370219-bib-0039] Terefe, H. , D. Wegary , Z. Bekeko , and M. Haile . 2023. “Distribution and Severity of Northern Leaf Blight (*Exserohilum turcicum*) in Major Maize Growing Areas of Ethiopia.” Ethiopian Journal of Agricultural Sciences 33, no. 2: 45–58.

[pei370219-bib-0040] Tilahun, T. , W. Dagne , D. Girma , and N. Meseret . 2012. “Maize Pathology Research in Ethiopia in the 2000s: A Review.” In Meeting the Challenges of Global Climate Change and Food Security Through Innovative Maize Research. Proceedings of the 3rd National Maize Workshop of Ethiopia, edited by M. Worku , S. Twumasi‐Afriyie , L. Wolde , et al., 193–201. EIAR/CIMMYT.

[pei370219-bib-0041] Wani, T. A. , G. N. Bhat , M. Ahmad , A. Anwar , and G. Zaffar . 2022. “Screening of Maize Germplasm for Turcicum Leaf Blight Resistance.” Journal of Applied and Natural Science 14, no. 2: 384–391. 10.31018/jans.v14i2.3337.

[pei370219-bib-0042] Welz, H. G. , and H. H. Geiger . 2000. “Genes for Resistance to Northern Corn Leaf Blight in Diverse Maize Populations.” Plant Breeding 119, no. 1: 1–14. 10.1046/j.1439-0523.2000.00462.x.

[pei370219-bib-0043] Wende, A. , S. Hussein , J. Derera , W. Mosisa , and M. D. Laing . 2013. “Preferences and Constraints of Maize Farmers in the Development and Adoption of Improved Varieties in the Mid‐Altitude, Sub‐Humid Agro‐Ecology of Western Ethiopia.” African Journal of Agricultural Research 8, no. 13: 1245–1254.

[pei370219-bib-0044] Yadesa, B. , and M. Diro . 2023. “Maize Lethal Necrosis Disease in Ethiopia: Status, Impact and Management Strategies.” Cogent Food & Agriculture 9, no. 1: 2183947. 10.1080/23311932.2023.2183947.

[pei370219-bib-0045] Yu, J. , J. B. Holland , M. D. McMullen , and E. S. Buckler . 2008. “Genetic Design and Statistical Power of Nested Association Mapping in Maize.” Genetics 178, no. 1: 539–551. 10.1534/genetics.107.074245. PMC2206100 18202393

